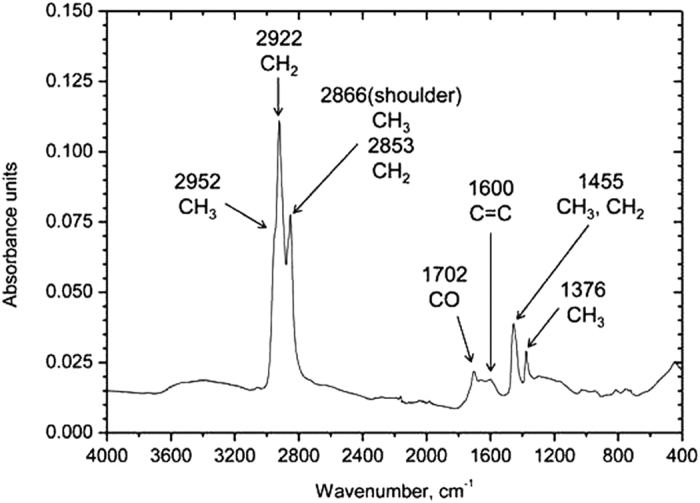# Nanodiamond Finding in the Hyblean Shallow Mantle Xenoliths

**DOI:** 10.1038/srep10765

**Published:** 2015-06-01

**Authors:** S. K. Simakov, A. Kouchi, N.N. Mel’nik, V. Scribano, Y. Kimura, T. Hama, N. Suzuki, H. Saito, T. Yoshizawa

**Affiliations:** 1 LLC “ADAMANT” Skolkovo Participant, Harchenko 19-A-7H, St.Petersburg, 194100, Russia; 2 Institute of Low Temperature Science, Hokkaido University, Sapporo 060-0819, Japan; 3 Lebedev Physics Institute, Russian Academy of Sciences, Leninsky pr. 53, Moscow, 119991, Russia; 4 Department of Biological, Geological and Environmental Sciences, University of Catania, Corso Italia Catania 55-I-95129 (Italy); 5 Department of Natural History Sciences, Faculty of Science, Hokkaido University, Sapporo 060-0810, Japan; 6 Research Division of JAPEX Earth Energy Frontier, Creative Research Institution, Hokkaido University, Sapporo 001-0021, Japan; 7 Equipment Management Center, Open facility Division, Creative Research Institution, Hokkaido University, Sapporo 001-0021, Japan

## Abstract

Most of Earth’s diamonds are connected with deep-seated mantle rocks; however, in recent years, μm-sized diamonds have been found in shallower metamorphic rocks, and the process of shallow-seated diamond formation has become a hotly debated topic. Nanodiamonds occur mainly in chondrite meteorites associated with organic matter and water. They can be synthesized in the stability field of graphite from organic compounds under hydrothermal conditions. Similar physicochemical conditions occur in serpentinite-hosted hydrothermal systems. Herein, we report the first finding of nanodiamonds, primarily of 6 and 10 nm, in Hyblean asphaltene-bearing serpentinite xenoliths (Sicily, Italy). The discovery was made by electron microscopy observations coupled with Raman spectroscopy analyses. The finding reveals new aspects of carbon speciation and diamond formation in shallow crustal settings. Nanodiamonds can grow during the hydrothermal alteration of ultramafic rocks, as well as during the lithogenesis of sediments bearing organic matter.

The formation of macroscopic diamonds is connected with deep upper mantle rocks, such as kimberlites and lamproites, formed at high P-T in the stability field of diamond. Micro-sized diamonds and rare ultra-high P-T minerals were found in shallow metamorphic rocks and ophiolite complexes, originated in the graphite stability field^1^,^2^. The formation mechanism of these metamorphic rock-hosted diamonds still represents a hotly debated topic^3^.

Nanodiamonds are carbon particles of enigmatic origin; they can be defined as the archetypal “macrosopic molecule”^4^. Hydrogen-terminated diamonds and nanometer-sized diamondoid hydrocarbons form a continuous structural series including lower diamondoids (<1 nm), higher diamondoids (~1 to 2 nm), nanocrystalline and chemical vapor deposition (CVD) diamonds (~2 nm to μm), and macroscopic diamonds (ref. 4). Nanosized diamond particles occur in chondrite meteorites and interplanetary dusts. These nanodiamonds are often associated with diamondoids^5^. Astronomers concluded that 3% of the carbon present in meteorites is in the diamond form. Using the telescope Spitzer, NASA scientists identified nanodiamonds in space in cold molecular clouds formed at low P-T. Moreover, astronomical observations suggest that 10–20% of interstellar carbon exists in the form of ultrananocrystalline diamonds^6^. Radio astronomy investigations detected hydroxyl OH, water and ammonia vapors, formaldehyde, carbon monoxide, methanol (alcohol), ethyl alcohol, and dozens of other more complex molecules in the clouds. In addition, Kouchi *et al.*^7^ related interstellar diamond formation with water and organic matter.

It is opportune to recall that the pressure of the diamond–graphite transition can be notably low for the formation of crystalline carbon nuclei, because surface energy is a significant part of their total thermodynamic potential at small sizes. In accordance with Jiang *et al.*^8^, at standard pressure and temperature conditions, diamond particles smaller than 6 nm are more stable than graphite ones of the same size. The stability of nanometer-sized diamonds increases when they derive from aromatic polycyclic hydrocarbons, since particles are hydrogen-terminated^9^. Experimental products synthesized by Kouchi *et al.* (ref. 7) from mixtures of interstellar organic matter and water at 150–400 °C and low pressure, confirm the formation of diamond traces. In addition, Simakov *et al.*^10^ reported the hydrothermal synthesis of nanodiamond particles with an average size of 70–80 nm from water-organic solutions at supercritical conditions (T = 500 °C, P < 1000 bar), considered a favorable environment for the diamond growth^11^.

The experimental and astrophysical data suggest that nanodiamonds may be found in Earth shallow crustal rocks, associated with the presence of organic matter and water. Accordingly, Manuella^12^ inferred that modern and fossil hydrothermal systems hosted in serpentinites, where organic matter and water coexist at a wide range of temperatures, can be considered as potential sites for the nucleation and growth of nanodiamonds.

On these grounds, a Hyblean serpentinized peridotite xenolith bearing asphaltene-like organic matter, which contain enigmatic clusters of tiny opaque carbonaceous flakes, was examined for the possible presence of nanodiamonds by using Raman spectroscopy and transmission electron microscopy (TEM). The results of this investigation are reported herein.

The sample is a strongly serpentinized and carbonated peridotite xenolith from a Tortonian tuff-breccia pipe located in the Hyblean Plateau (Sicily, Italy). Hyblean tuff-breccia deposits bear a xenolith suite, which represents direct evidence for the unexposed lithospheric roots of this Central Mediterranean area, consisting of the relict of the ultraslow-spreading Mesozoic Ionian-Tethys Ocean^13^,^14^,^15^. Most of Hyblean xenoliths contain evidence of abyssal-type hydrothermal metasomatism, including the serpentinization of ultramafic rocks. In addition, traces of hydrocarbons were found in some hydrothermally altered gabbro and serpentinized ultramafic xenoliths^16^,^17^. These hydrocarbons were interpreted as abiotic organic compounds produced in the serpentinite basement (ref. 15) via Fischer–Tropsch-type (FT-t) reactions^18^:
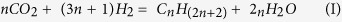
there 1 < = n < + ∞

Serpentinized peridotites consist of coarse polycrystalline aggregates of pyroxenes (enstatite and Cr-Al-diopside) and Cr-Al-spinel immersed in a dominant assemblage of secondary minerals primarily consisting of calcite and serpentine (lizardite) with minor talc, smectite, magnetite, trevorite, millerite, pyrite, Fe-sulfate, and Sn- and Pb- sulfides. The mineral chemistry and whole rock chemical composition (major and trace elements) were reported by Scirè *et al.* (ref. 17). The spinel occurrence among the relict primary minerals suggests that, in its earliest geologic history, the sample equilibrated in the upper mantle in the spinel-peridotite stability field, where graphite is the most stable carbon allotrope.

Notably, the sample contains ~5 vol.% of S-bearing asphaltene-like organic matter intermixed with serpentine fibers, forming discrete blebs and filling narrow cracks in the pyroxene grains and between grains. Furthermore, transmitted light observations revealed the abundance of micrometer-sized black carbonaceous flakes (Fig. 1) of enigmatic origin interspersed in the hydrocarbon blebs.

The different generations of serpentine veins in the sample, along with their relationships with Fe-oxide and Fe-Ni sulfide micrograins, suggest that serpentinization developed in several stages at different temperatures; hence, the *f*(O_2_) of the system evolved from reducing (i.e., below the quartz-fayalite-magnetite buffer) to significantly oxidizing conditions (e.g., above the magnetite-hematite buffer), with the water/rock ratio increasing concomitantly through time. The sulfur fugacity was probably in the range of −1.8 < log*f*(ΣS) < −0.6, as suggested by the sulfide stability fields reported by Frost^19^. Moreover, an increasing *f*(CO_2_) through the geologic history of the sample is recorded by its extensive and pervasive carbonation (Fig. 1), which probably developed in different stages (ref. 17). The serpentinization of the olivine, which produced lizardite polytype, is compatible with T ≤ 300 °C as suggested by experimental results at P ≤ 0.1 GPa and thermodynamic modeling^20^,^21^.

Scirè *et al.* (ref. 17) described the multistage geologic history of the sample on the basis of its mineral assemblage and studied the organic matter occurring therein. To summarize, an upper mantle volume consisting of nominally anhydrous spinel-bearing mantle peridotite veined by clinopyroxenite was tectonically exhumed at a shallow sub-seafloor area of the slow-spreading Ionian-Tethys Ocean in the Early Triassic. There, ultramafic rocks interacted with seafloor-dominated hydrothermal fluids. The serpentinization process developed through time under different physicochemical conditions; since its early stages, the conditions favored the formation of abiogenic hydrocarbons via FT-t synthesis. Fe-Ni alloys (e.g., awaruite) were putative catalysts of the FT-t synthesis, which, at a molecular scale, can be viewed as a catalyst-surface process^22^. The fate of the awaruite catalyst from the sample was oxidation to trevorite in the final stages of the serpentinization process.

The subsequent stages in the history of the sample’s alteration were characterized by its incipient steatization, formation of mafic clays after serpentine, and severe carbonation of hydrocarbons and silicate minerals; these processes occurred at increasing *f*(O_2_) and *f*(CO_2_) conditions, and hence, decreasing *f*(H_2_) conditions. Scirè *et al.* (ref. 17) suggested that at the final stage of serpentinization, the FT-t synthesis also yielded unsaturated hydrocarbons, including aromatics rings whose polycondensation gave rise to asphaltenes. In the Late Miocene, the sample was entrained as a xenolith in a fluid-dominated diatreme structure and hence erupted in a shallow marine setting^23^.

## Results

The asphaltene samples were treated with strong acids and organic solvents to remove silicates, carbonates, sulfides, and organics, and micro Raman observations were then performed. The micro Raman spectra show clearly visible bands at 1300 and 1600 cm^−1^, indicating the presence of nanodiamonds and nanographite (Fig. 2A). Figure 2B shows the distribution of nanodiamonds and graphites observed through the microscope of the Raman spectrometer. Note that the size of a CCD pixel (~1.2 μm) is larger than that of a nanodiamond aggregate (Fig. 3a).

The black carbonaceous flakes (untreated asphaltene) were observed using a high-resolution transmission electron microscope (HRTEM). Figure 3a shows a TEM image and an electron diffraction pattern of the red circle region, clearly indicating that the grain is a polycrystalline diamond. The atomic composition of the diamonds is 99.6% carbon with 0.4% oxygen (Fig. 3b). A HRTEM image of the sample shows that this grain is an aggregate of nanodiamonds ranging from 1 to 6 nm (Fig. 3b); the dominant size is ~6 nm, and a few 10 nm-sized diamonds are observed. The image also reveals that graphite with a thickness of 10–20 layers is only observed at the rim of the nanodiamond aggregate (Fig. 3d). This is the first clear evidence of the occurrence of nanodiamonds in a serpentinite. The infrared spectrum of residual black carbonaceous materials, without silicates and soluble organic materials (treated asphaltene), shows that aliphatic hydrocarbons (CH_3_ and CH_2_) are more abundant than aromatic compounds (C = C; Fig. 4). The occurrence of the small C = O peak at 1704 cm^−1^ suggests that this sample never experienced temperatures above 330 °C^24^.

The stable carbon isotope values (δ^13^C) of the saturated and aromatic hydrocarbon fractions extracted from the asphaltene sample were −29.8 and −32.8‰, respectively. The δ^13^C of the remaining solid residue (treated asphaltene) was −29.0‰.

## Discussion

Recalling the aforementioned concepts, the stability of nanodiamonds in the graphite field is enhanced by their surface energy, and depends on the critical size of the particles that decreases with increasing temperature (ref. 8). On this basis, nanodiamonds found in the studied Hyblean serpentinite xenolith have sizes of 6 and 10 nm, which are compatible with two temperatures of 447 °C and 117 °C, respectively. These temperatures were estimated by using the size-temperature equation extracted from Manuella (ref. 12). Similar values derive from some petrologic evidence: (i) the presence of lizardite (ref. 17) (suggests that the highest serpentinization temperature was ~300 °C^20^; (ii) the FT-IR spectrum obtained by the ATR method for the residual black carbonaceous materials (Fig. 4) indicates temperatures lower than 327 °C. Therefore, we propose that the temperature of nanodiamond formation during serpentinization may correspond to the range of 150–300 °C.

With regard to serpentinization, we consider the following reaction for olivine hydration and formation of serpentine along with magnetite and dihydrogen.
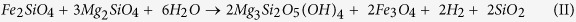


Taking into account the xenolith mineral assemblage and the phase relations reported by Evans *et al.* (ref. 20), we suggest that the initial *f*(O_2_) was near the quartz-fayalite-magnetite (QFM) buffer. In fact, the lack of primary high metal/S Ni-Fe sulfides (e.g., pentladite) in the sample suggests an early desulfurization event with the probable formation of Ni-Fe alloys (e.g., awaruite; see previous section). This stage of the process is related to the abiotic hydrocarbon synthesis via FT-t reaction. If we consider the CO_2_-SiO_2_-FeO-H_2_O system, the following reaction is also possible.
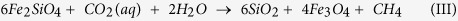


During the development of reactions (II and III), *f*(H_2_) increases and *f*(O_2_) decreases, corresponding to the transition from QFM to würtzite-magnetite (WM) buffer.

To estimate the composition of the C-O-H fluid system from which free carbon precipitated, we used the GFluid program^25^. A water-methane-rich fluid forms at conditions near the QFM buffer, and a methane-rich fluid occurs at WM buffer. Diamond nuclei formation is possible from H_2_O-CO_2_-rich fluids containing minor CH_4_ component^26^, which corresponds to the region of *f*(O_2_) between CCO (upper limit of carbon stability) and QFM buffers. Rudenko *et al.*^27^ indicated that diamond may crystallize under such chemical conditions by the reaction of methane and carbon dioxide:
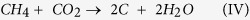


Furthermore, it is opportune to note that the new carbon isotopic results gained in this study are consistent with the isotopic data of methane formed in hydrothermal systems. Indeed, δ^13^C values retrieved from the analyzed xenolith-bearing hydrocarbons are similar to those measured by Etiope *et al.*^28^ in a gaseous hydrocarbon seepage from serpentinizing ultramafic rocks in the Othyris Ophiolite complex (Greece), where the δ^13^C values range between −32 and −30%. This comparison confirms the abiotic hydrocarbon source of the nanocarbon formation in the Hyblean serpentinite xenolith.

**Figure 1:**
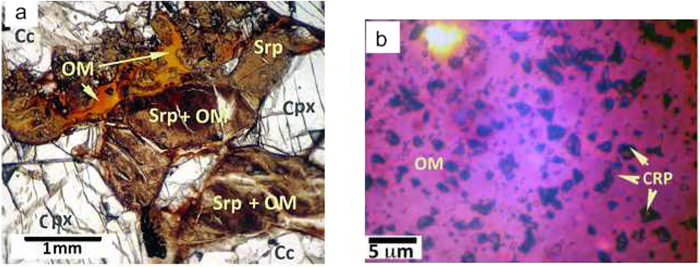
**a**) Portion of a thin section of the sample viewed under an optical microscope (plane-polarized transmitted light). Serpentine replaces former olivine, whereas organic matter (purplish) occurs between serpentine fibers or as discrete blebs. **b**) Thin section highlighting clusters of black carbonaceous particles immersed in the purplish organic matter (plane-polarized transmitted light). Legend: OM = organic matter; CPR = micrometric clusters of carbonaceous particles. Cpx = clinopyroxene; Srp = serpentine and coexisting talc, chlorite, and smectites; Cc = calcite.

**Figure 2:**
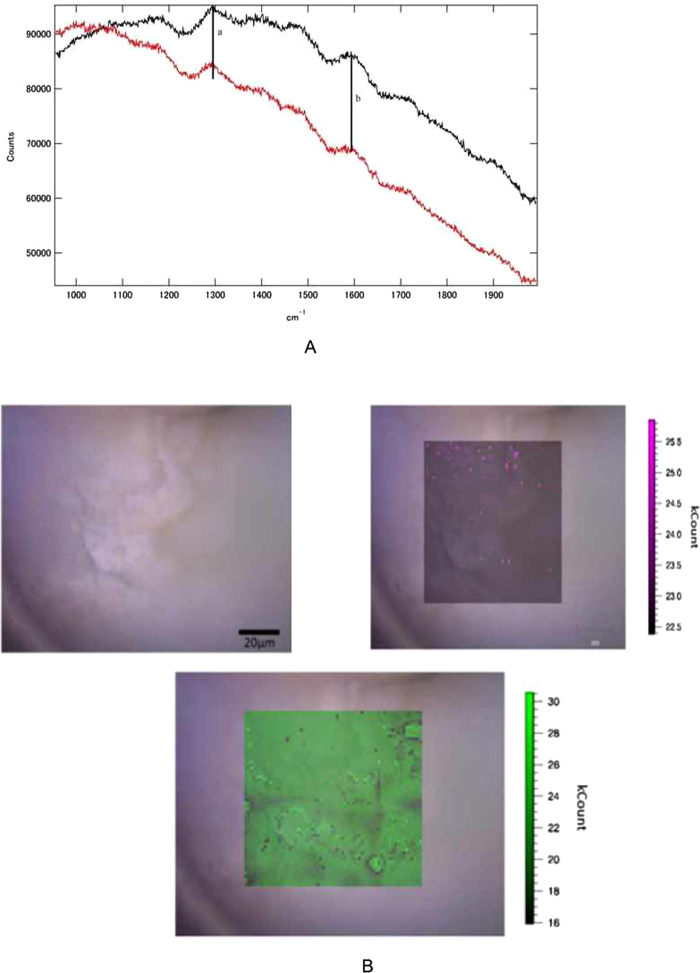
**a**) Raman scattering spectra of treated (black) and untreated (red) asphaltene sample obtained on a Raman microscope: **a**) 1300 cm^−1^, indicating the presence of nanodiamonds; **b**) 1600 cm^−1^, indicating the presence of graphitic planes. **b**) Optical microscopy image and corresponding micro-Raman images of the remaining solid residue (treated asphaltene) observed at 1300 cm^−1^ (green) and 1600 cm^−1^ (purple), showing the distributions of naodiamonds and graphite, respectively. Respective micro-Raman images overlap on the optical microscopy image. It is noted that the size of nanodiamonds aggregate is smaller than that of a CCD pixel (1.2 μm) for Raman imaging.

On the other hand, the serpentinization reaction developing near the WM buffer resulted in the full replacement of olivine, partial replacement of orthopyroxene, and formation of hydrocarbons (mostly alkanes) along with the majority of the free nanocarbons (nanodiamond and graphite). The 6-nm diamonds probably formed in this stage of the serpentinization process. As a result, carbon nanoparticles were immersed in the hydrocarbon medium (Fig. 1), and protected by the later stage, corresponding to the carbonation. The later stage of the xenolith hydrothermal history involved carbonate formation at the expense of the previously formed hydrocarbons (i.e., the oxidative mineralization of the organic carbon). The carbonation process began at T < 150 °C, when the olivine was completely replaced by serpentine and the generation of H_2_ was completed, while the flushing of relatively oxidized seawater with dissolved CO_2_ increased. Therefore, oxygen fugacity grew dramatically during the transition from WM to hematite-magnetite (HM) buffer, and oxidative degradation of hydrocarbons occurred. The 10-nm diamonds possibly crystallized during the latter stage of alteration (carbonation).

## Conclusions

The discovery reported here deals with the problem of the diamond formation in the Earth crust rocks and oceanic lithosphere (ref. 1, 2, 3). Our results confirm that nanodiamonds can form during serpentinization in the stability field of graphite, from an organic-water system (ref. 12), similar to that occurring in some meteoritic parent bodies (ref. 7,24).

Considering that the physicochemical conditions of nanocarbon formation inferred from our results are similar to those observed in the final stage of sediment lithogenesis^29^, we speculate that nanodiamonds may also occur in some sedimentary rocks bearing organic matter. Our results further suggest that nanodiamond formation is possible during the oxidative degradation process of crude oils, together with the formation of macromolecular hydrocarbons as well as bitumens, anthraxolites, and asphaltenes.

Our finding also promotes the hypothesis of prebiotic reactions during the exothermic serpentinization processes^30^. Indeed, the hydrogenated nanodiamonds create crystalline water layers, which are an excellent medium for the rapid transport of positive charge in the form of hydrogen protons. This provides the anomalously high proton mobility in bulk water, which exceeds even that of fast solid-state proton conductors^31^. In this respect, the proton gradients in alkaline hydrothermal vents are viewed as key processes in the origin of prebiotic molecules.

## Methods

To remove silicates, calcite, sulfides, and soluble organic materials from black carbonaceous flakes, the asphaltene sample was treated with strong acids (HCl and HF) and organic solvents (methanol, acetone, and benzene). We refer to the remaining solid sample as treated asphaltene, and the original sample is termed untreated asphaltene.

**Figure 3:**
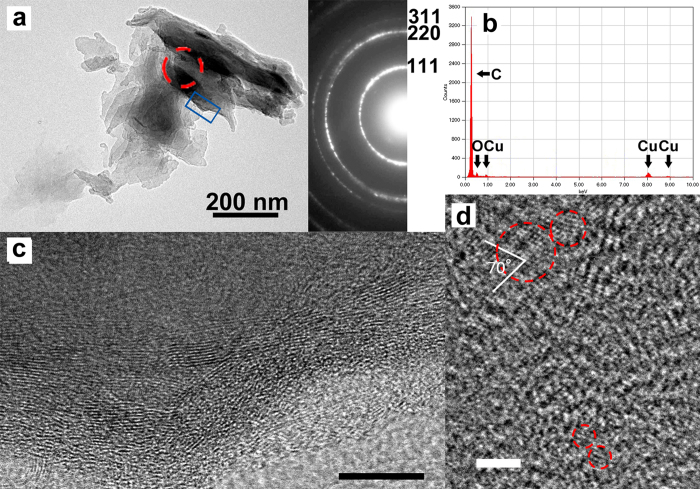
Transmission electron microscope images of carbonaceous grains in asphaltene-bearing serpentinite. (**a**) Low-magnification- bright-field image and selected area (red circle) electron diffraction pattern. Diamonds are clearly identified in the diffraction pattern. (**b**) Energy dispersive X-ray spectrum of the sample showing that the composition of carbonaceous grains is pure carbon with small amount of oxygen (C: 99.6%, O: 0.4 atomic %, no nitrogen). Note that the Cu peaks are from the sample grid. (**c**) HRTEM image of the blue square region showing graphite lattice fringes of (002), which were seen only in the rim of the grain. The scale bar is 10 nm. (**d**) Typical HRTEM image of several nanometer-sized diamonds with crossed lattice fringes of (111). The scale bar is 2 nm.

The Raman equipment was a Renishaw Raman imaging microscope consisting of a single spectrograph (0.25 m focal length) containing a holographic grating (1200 grooves mm^–1^), an Leica microscope and a Peltier-cooled CCD detector. The spectra and image were excited with 785 nm radiation from a 50 mW air-cooled laser and the laser beam was focused on the sample by a X50 lens to give a spot size of ca. 1 μm; the resolution was better than 1 cm^–1^. We employed a HRTEM (JEM-2100F, JEOL) located at the Tohoku University, Sendai, Japan. The TEM with a field-emission electron gun was operated at an accelerating voltage of 200-kV and had equipped with an energy dispersive X-ray spectrometer (EX-2300, JEOL). Samples for TEM observations were prepared by crushing the untreated asphaltene. The sample shown in Fig. 3 was analyzed using a JEOL EX-2300. No peaks other than that of carbon and little amount of oxygen originating from the sample were detected, even when the electron beam was focused on other locations in the particles. To measure the IR spectra of the treated asphaltenes, an FTIR spectrometer with ATR (Spectrum one/Universal ATR, PerkinElmer) was used.

Soluble organic matter was extracted from untreated asphaltene with dichloromethane. The saturated and aromatic hydrocarbon fractions were obtained by silica gel column chromatography (Silica gel 60, Merck). Stable carbon isotopic analyses for the saturated and aromatic hydrocarbons were performed using gas chromatography (GC)-combustion-isotope ratio mass spectrometry (Agilent 6890A GC and Finnigan Delta V Advantage), equipped with a fused silica DB-5HT column (30 m × 0.32 mm i.d., J&W). The GC oven temperature was initially programmed to 40 °C and held isothermally for 2 min; it was then programmed to 300 °C at 4 °C min^−1^ and held isothermally for 20 min. The combustion of hydrocarbons was performed in a micro-volume ceramic tube with CuO, NiO, and Pt wires at 900 °C. Stable carbon isotope data are reported as δ^13^C values relative to the VPDB scale. The δ^13^C values of total saturated and total aromatic hydrocarbons were determined by comparison with the Oztech isotope ratio reference gas (δ^13^C value = −40.80% VPDB) (Shoko Co. Ltd.). The analytical precision of δ^13^C value was ± 0.4%. The δ^13^C of the remaining solid residue (treated asphaltene) was analyzed by elemental analyzer-isotope ratio mass spectrometry (EA1112-Delta V Advantage ConFloIV System, Thermo Fisher Scientific) at SI Science Co. Ltd., Japan.

## Additional Information

**How to cite this article**: Simakov, S. K. *et al.* Nanodiamond Finding in the Hyblean Shallow Mantle Xenoliths. *Sci. Rep.* **5**, 10765; doi: 10.1038/srep10765 (2015).

## Figures and Tables

**Figure 4:**